# Topical capsaicin 8% patch in peripheral neuropathic pain: Efficacy and quality of life

**DOI:** 10.1177/20494637231201502

**Published:** 2023-09-12

**Authors:** Márcia Pitrez Santos, Francisco Lemos, Joana Gomes, José Manuel Romão, Dalila Veiga

**Affiliations:** 1 89239 Medicine Faculty, Instituto de Ciências Biomédicas Abel Salazar – University of Porto, Porto, Portugal; 2 Department of Anesthesiology, 112085 Centro Hospitalar Universitário de Santo António E.P.E., Porto, Portugal

**Keywords:** Capsaicin, neuropathic pain, effectiveness, safety, quality of life

## Abstract

**Introduction:**

Peripheral neuropathic pain (PNP) is one of the most challenging diseases to treat with a significant negative impact on the patients’ health-related quality of life (HRQoL). Capsaicin 8% patch has arisen in the last decades as an alternative to oral drugs in the treatment of PNP with fewer side effects and promising results in efficacy.

**Objectives:**

This work aims to evaluate the effectiveness of the topical application of capsaicin in PNP and its impact on patients’ HRQoL based on the use of capsaicin in a tertiary hospital of Oporto.

**Methods:**

This study included 100 patients with localized PNP with poor pain control and without improvement with previous treatments that were treated at least once with an 8% capsaicin patch. Effectiveness on pain relief, number of treatments needed, safety and impact on HRQoL were assessed through a set of questionnaires.

**Results:**

Regarding the aetiology of PNP, 67.6% (*N* = 46) have post-surgery or trauma induced PNP with 64.7% (*N* = 44) of patients reporting pain in the lower limb. After the treatment, 30.9% (*N* = 21) felt minimally improved, 22.1 (*N* = 15) felt much improved and 13.2 (*N* = 9) felt very much improved. On a scale from 1 to 10, in the week prior to the survey, the median intensity of pain was 6 and the median interference in quality of life was 7. The majority of patients still report limitations in mobility and daily activities and moderate pain.

**Conclusion:**

Capsaicin 8% patch is effective in PNP treatment at least in the short term. Repeated applications may be important for long-term analgesia. The low systemic dose and few side effects mean that the treatment is generally well tolerated by patients. Due to the analgesic effect, capsaicin can improve the HRQoL of patients with PNP.

## Introduction

Neuropathic pain is defined by the International Association for the Pain Study as ‘*pain caused by a lesion or disease of the somatosensory nervous system*’.^1^ The somatosensory system includes both the peripheral neurons and the central nervous system. The affected fibres in the peripheral nervous system are mainly the small unmyelinated C fibres and the myelinated A fibres, predominantly the Aβ and Aδ fibres.^2,3^ The damage to these fibres provokes loss of function and paradoxically an increase in pain sensitivity and spontaneous pain – peripheral neuropathic pain (PNP).^1^ Although the pathophysiology is not fully understood, this paradoxical effect of peripheral neuropathy is due to a dysfunction of transmission of sensory signals from the periphery to central neurons.^3,4^ Imbalance between excitatory and inhibitory signalling, ion channel alterations and altered modulation of pain stimuli in the CNS seem to be the mechanisms responsible for PNP by maintaining a state of hyperexcitability.^3–5^ Besides, remains of an injured afferent nerve can generate ectopic activity causing pain in a numb region.^3–5^

Chronic PNP is defined as pain due to injury of the peripheral sensory fibres that is persistent or recurrent pain lasting 3 or more months.^1^ The transition to chronic pain is likely due to a series of alterations, occurring from the periphery to the brain cortex over time.^3,4^ This disease has several different aetiologies such as diabetes mellitus or other metabolic dysfunctions, peripheral nerve injury, painful polyneuropathy, post-herpetic neuralgia or other infectious diseases (HIV), painful radiculopathy after chemotherapy, immune dysfunctions (Guillian-Barré Syndrome), inflammatory disorders (trigeminal neuralgia), inherited neuropathies and other specified and unspecified causes.^1,3,5–7^ It has an estimated prevalence of 6.9% to 10% worldwide.^1,3,8,9^ In general, this condition is more frequent in the elderly (>60 years) and women rather than in men.^3,6,10^ Also, the lumbar and lower limbs and neck and upper limbs are the most affected locations.^3,6^ It is expected to increase in the future due to higher rates of conditions that affect the small fibres such as diabetes mellitus, chemotherapy with higher survival rates and overall ageing of the global population.^3^

PNP is experienced differently from patient to patient, even the ones with the same underlying pathology.^1,7^ It can be described as different sensations like burning, pricking, shooting, pins and needles, freezing pain and squeezing. It is often present electric-shock-like pain either isolated or associated with ongoing pain. Pain may be spontaneous or evoked when it surges in response to a painful stimulus (hyperalgesia) or a usually non-painful stimulus (allodynia).^1,3–7^ Pain can be associated with other positive symptoms like abnormal sensations either unpleasant (dysaesthesia) or pleasant (paraesthesia).^1–3,7^ Also, there are often negative symptoms such as decreased or loss of sensation.^1–3^

Regarding the health-related quality of life (HRQoL) in patients with chronic PNP, there is a strong negative impact on patients’ lives with great suffering and disability.^1,3,5,8,11,12^ HRQoL is considerably poorer in individuals with chronic neuropathic pain than in those without pain and even when compared to patients with chronic pain of non-neuropathic characteristics.^8,13–15^ Spinal neurons have projections to the thalamus and cortex and parallel pathways to the limbic areas, that in PNP are altered. This contributes to high pain, anxiety, depression and sleep problems described in the majority of patients with chronic PNP.^3,4^ Additionally, symptoms tend to persist and respond less to medication becoming chronic, compromising patients’ physical and mental health.^3,15,16^ For these reasons, PNP has a detrimental effect on HRQoL.

Treatment of PNP consists of symptom management once the aetiology is not usually treatable. The current pharmacotherapy for PNP is systemic medication in monotherapy like gabapentinoids (pregabalin and gabapentin), tricyclic antidepressants (amitriptyline) and serotonin noradrenaline-reuptake inhibitors (duloxetine).^4,12,17–20^ The most frequent second-line option is the association of gabapentinoids and TCAs or SNRIs.^17,20^ Strong opioids are not recommended for chronic PNP as a first-line therapy.^3,4,19,21^

However, these drugs have limited efficacy in this pathology contributing only to modest pain relief but have potentially serious common side effects like somnolence and cognitive impairment.^3,4,12,19,20^ Besides, the majority of patients with PNP are older and frequently poli-medicated.^3,4,19,20^ For these reasons, there has been an increase in the use of topical treatments like local anaesthetics (lidocaine 5% patch) and capsaicin (8% patch), currently second or third-line treatments for PNP.^17–21^

Capsaicin is an alkylamine (trans-8-methyl-N-vanillyl-6-none amid) of natural origin present in chilli peppers and other plants of the capsicum plant family.^18,22^ This molecule is a highly selective agonist of transient receptor potential vanilloid 1 (TRPV1) and induces analgesia through the defunctionalization of TRPV-1-expressing nociceptors. TRPV-1 is a non-selective ion channel receptor complex, present on skin nociceptive afferents, mostly in C fibres and some Aδ, particularly the ones responsible for painful or noxious sensations.^18,22–26^ TRPV1 has been proven a key molecule in peripheral hyperexcitability responsible for neuropathic pain. When activated, TRVP1 allows the influx of sodium and calcium that originate the electrical impulse.^12,18,23,24,27^

A recent study proposes that the associated analgesia mechanism of capsaicin is due to the accelerated regeneration of peptidergic fibres and restoration of function rather than the destruction of these fibres. A disease-modifying effect of capsaicin on these fibres already occurs 4 weeks after treatment and repeated application of capsaicin might enhance the regenerative properties of peptidergic fibres, which might explain sustained and long-lasting pain relief.^28^

Capsaicin is available in two different formulations according to its concentration: low-concentration skin cream (0.025%–0.1% capsaicin) or high-concentration skin patch (8% capsaicin, 179 mg).^18,22,29^ Low doses of capsaicin induce desensitization of nociceptors reversible in hours owing to inactivation and inhibition of TRVP1 without causing structural changes. Alternatively, high doses of capsaicin induce defunctionalization for weeks that include loss of membrane potential and retraction of epidermal nerve fibre endings.^18,22^ Overall, this long-lasting effect is caused by structural ablation of TRPV1-expressing afferent terminals generally leading to a reduction of epidermal nerve fibre density (ENFD).^12,18,22–24,26,30^ It is important to note that is not obligatory to have nerve fibre degeneration to attain analgesia. The intrinsic effect of capsaicin on TRPV1 receptors can be enough to cause alteration in nociception.^31,32^ Reduction of ENFD does not necessarily guarantee decreased reaction to mechanical stimuli nor analgesia for PNP.^18^ The impaired nociceptor functionality is responsible for reduced warmth detection, flare response and laser-evoked heat pain, without affecting tactile sensitivity or cold pain.^33,34^

Besides its action in impulse transmission, capsaicin causes depletion of substance P, a neuropeptide involved in nociceptive transmission and inflammation; however, its effect is not sufficient to produce analgesia on its own.^18,22^ Also, capsaicin induces TRPV1+ neurons to release somatostatin responsible for analgesia. This mechanism is thought to contribute to the reduction of neuropathic pain at least in the initial period. ^18,20,22,35^

Capsaicin is well absorbed in the skin with minimal systemic concentration minimizing the probability of systemic adverse effects and does not interfere with the metabolism of other drugs because it avoids first-pass metabolism.^11,18,26,29,36–39^ Capsaicin adverse effects are few and mild. It has been reported a small transitory increase in blood pressure and local reactions such as burning pain, erythema and pruritus.^12,18,22,23^ Nevertheless, the low dose and transient side effects allow for treatment with capsaicin 8% patch to be well tolerated in repeated applications.^11,26,36,38–41^

Multiple studies demonstrated that capsaicin 8% plaster is effective in controlling pain intensity for several weeks at a time and reducing the painful area in patients with PNP from various aetiologies, even in slower responders. Furthermore, the majority of patients reported an improvement in their quality of life. ^11,29,36–38,40–43^

This treatment has been performed at Centro Hospitalar e Universitário de Santo António (CHUdSA) since 2012, relying exclusively on hospital application and currently one of the first-line therapeutics for PNP. This article aims to evaluate the effectiveness of the topical application of capsaicin in peripheral neuropathic pain and its impact on patients’ quality of life based on published literature and the use of capsaicin in a tertiary hospital of Oporto.

## Methods

A retrospective study was elaborated at a tertiary hospital of Oporto with approval by the Ethics Committee and the Department of Education, Training and Research and the Responsible for Clinical Information Access (Ref. 2022.203). This study included 100 patients with localized PNP with poor pain control and without improvement with previous treatments that were treated at least once with an 8% capsaicin patch in this hospital. Of these, 9 were excluded due to death and 23 did not respond to the telephone interview. Written informed patient consent was obtained previously.

The following information was consulted in the medical files and/or inquired about through the telephone survey:

- age and gender,
- telephone number,
- diagnostic,
- duration of pain,
- number of treatments administrated in CHUdSA,
- location of pain,
- pain characteristics through the application of the validated Portuguese version of the “Neuropathic Pain Questionnaire” (DN4) (adapted),
- pain response to treatment immediately after the treatment through the application of the validated Portuguese version of the ‘Patient Global Improvement Change Scale’ (PGIC) and
- pain evaluation and interference in quality of life through the application of the validated Portuguese versions of ‘Brief Pain Inventory’ (BPI) (adapted) and ‘EuroQol Questionnaire, 5 dimensions, 3 levels’ (EQ-5D-3L).

The telephone survey was undertaken by the first author between February and April 2023.

Table 1 presents the description of the categories considered for the analysis of the variables under study.

**Table 1.** Characterization of variables.

Variable	Codification
Pain aetiology	Amputation pain
	Diabetic polyneuropathy
	Post-surgical or trauma pain
	HIV-neuropathy
	Chemotherapy neuropathy
	Trigeminal neuralgia
	Post-herpetic neuralgia
	Other
Pain location	Face
	Cranium
	Cervical region
	Superior limb
	Dorsal region
	Thoracic region
	Abdominal region
	Lumbar region
	Pelvic region
	Inferior limb
BPI – In the last week, how effective were the medications you used to relieve your pain?	Nothing
	A little
	More or less much
	Completely
BPI – In the last week, how frequently do you felt pain after taking medication?	Always
	Frequently
	Sometimes
	Rarely
	Never

### Pain assessment

The DN4 questionnaire was originally developed and applied in France with the purpose of screening for neuropathic pain. Nowadays, DN4 questionnaire is used worldwide and is validated for the Portuguese population. It includes 10 items related to pain characteristics, from which 3 are descriptive terms for pain quality (burning sensation, painful cold sensation and electric shocks sensation), 4 refer to abnormal sensations associated with the pain (tingling, pricking, numbness and itching) and the rest (touch hypoesthesia, prick hypoesthesia and touch allodynia) is evaluated in the physical examination of the painful area. In this study, were used only the first 7 items.^44^ For the statistical analysis, a positive answer to any item is coded as a value of 1 and a negative item is coded as a value of 0.

To assess pain responsiveness to the capsaicin treatment was used the PGIC questionnaire. Literature defends that PGIC significantly correlates with pain severity, pain interference in QoL and treatment efficacy.^45^ It consists of a 7-point score from ‘very much improved’ to ‘very much worse’ according to the patient’s perception of pain changes after the treatment; however, it does not indicate in what aspect the pain improved. This questionnaire has been validated by IASP.^45^

The BPI questionnaire includes 15 items to evaluate the existence of pain, severity, localization, functional interference and efficacy of therapeutic strategies, in the last week. In this study, was applied an adapted version contemplating the following variables: pain severity scale with four items (maximum, minimum, mean and in the moment) using a scale from 1 to 10, pain interference scale with 7 items (general activities, mood, mobility, work, personal relations, sleep and life pleasure) using a scale from 1 to 10, and 2 items to assess the efficacy of current pharmacological treatment. This questionnaire has been validated by IASP given its reproducibility and sensitivity in pain diagnosis, follow-up, and characterization. It also was translated, adapted and validated for the Portuguese population.^44^ For statistics purposes, the severity of pain was evaluated using the median of the 4 four items and the pain interference was also evaluated using the median of the 7 items.

### Health-related quality of life

Regarding the evaluation of the health-related quality of life in this set of patients was applied the EQ-5D-3L questionnaire. This instrument is used to evaluate the quality of life related to health status through a system composed of five dimensions (mobility, personal hygiene, daily activities, pain/malaise and anxiety/depression) with three levels each (no problems, few problems and extreme problems) according to patient’s perception. It has been validated for the Portuguese population.^46^

Statistical analysis was performed using IBM SPSS Statistics®, version 28.0.1.0.

## Results

Of the 68 individuals who responded to the telephone survey, 77.9% (*N* = 53) are female and 22.1% (*N* = 15) are male. (Table 2) The age gap in this group is from 30 to 79 years old with an average of 61.4 years. (Table 3) Our non-response rate was of 22.1% (*n* = 32).

**Table 2.** Gender characterization.

Gender	Frequency
Female	53 (77.9%)
Male	15 (22.1%)

**Table 3.** Age characterization.

Mean	61,46
Median	64,00
Standard deviation	12,068
Interval	49
Minimum	30
Maximum	79

Regarding the aetiology of PNP, 67.6% (*N* = 46) have post-surgery or trauma induced PNP. Other/non-determined aetiologies correspond to 19.1% (*N* = 13) of the patients. 4.4% (*N* = 3) patients refer post-herpetic neuralgia, 1.5% (*N* = 1) have diabetic polyneuropathy and 1.5% (*N* = 1) have pain in the residual limb after amputation. (Table 4)

**Table 4.** Aetiology characterization.

Aetiology	*N* (%)
Amputation pain	1 (1.5)
DNP	1 (1.5)
Post-surgical or trauma pain	46 (67.6)
HIV-neuropathy	0 (0)
CINP	4 (5.9)
Trigeminal neuralgia	0 (0)
Post-herpetic neuralgia	3 (4.4)
Other	13 (19.1)
Total	68 (100)

**Figure 1.**
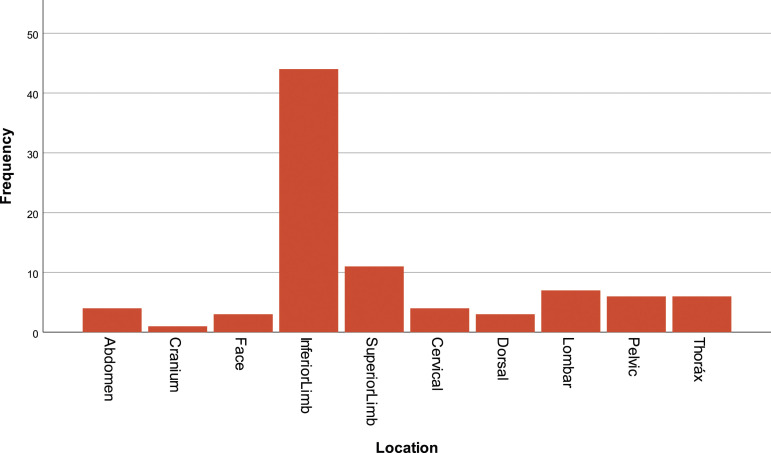
Pain location.

The most frequent location for PNP in this group is the lower limb, 64.7% (*N* = 44), followed by the upper limb, 16.2% (*N* = 11), and the lumbar region, 10.3% (*N* = 7). Other locations include the pelvic region, 8.8% (*N* = 6), thorax, 8.8% (*N* = 6), cervical region, 5.9% (*N* = 4), abdomen, 5.9% (*N* = 4), face, 4.4 (*N* = 3), and cranium, 1.5% (*N* = 1) [insert Figure 1].

**Figure 2.**
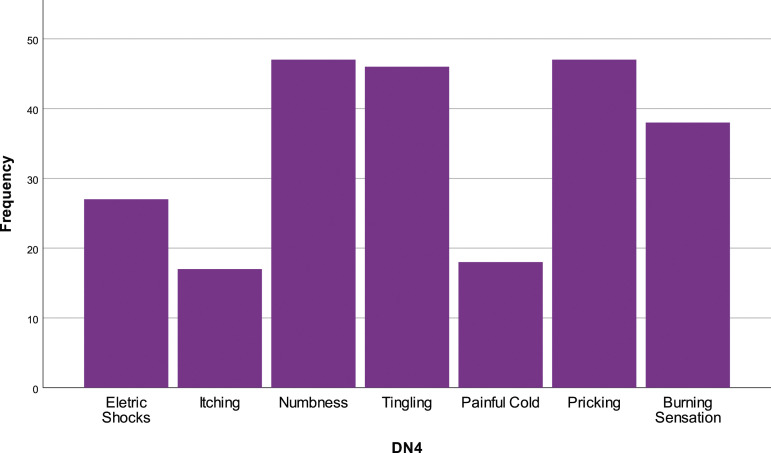
Pain characteristics (DN4 questionnaire).

In the DN4 questionnaire, 72.3% (*N* = 47) of the individuals refer to pins and needles, 72.3% (*N* = 47) to numbness, 70.8% (*N* = 46) to tingling and 26.2% (*N* = 17) to itching. The most frequent quality attributed to pain was burning, 58.5% (*N* = 38), next was electric shocks, 41.5% (*N* = 27) and 27.7% (*N* = 18) referred to painful cold [Figure 2].

**Figure 3.**
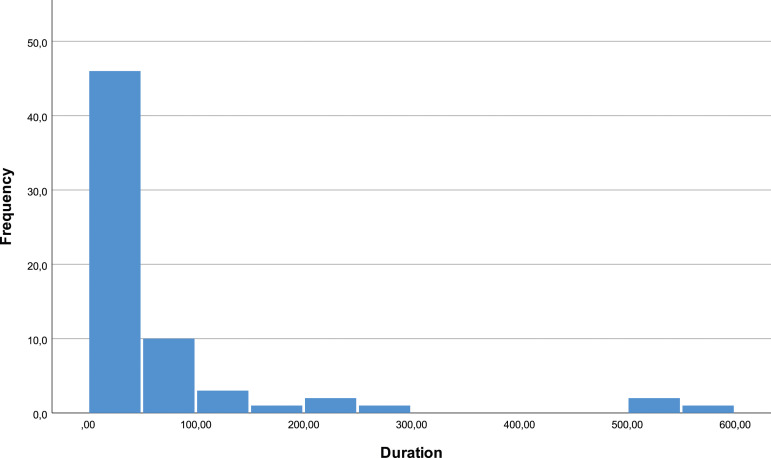
Pain duration.

**Figure 4.**
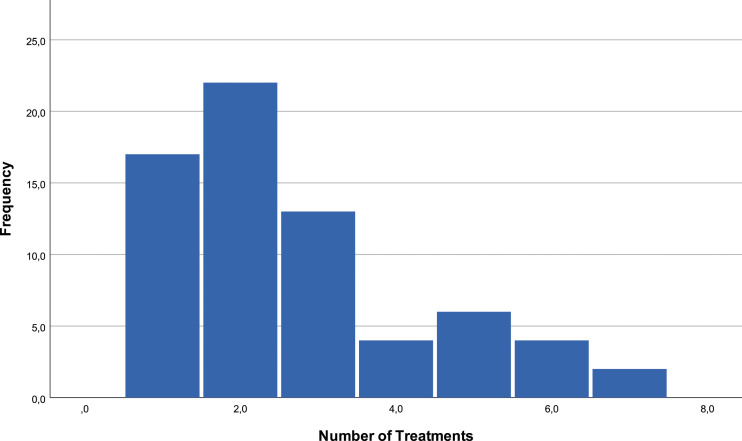
Number of treatments.

The duration of pain until the first treatment varies from 2 months to 49 years, with an average of 71.36 months and a median of 30 months. 25% of patients have a duration of pain inferior to 12 months, but 75% have a duration over 61.5 months. The median of the number of treatments is two, with an interval of values between 1 and 7 [Figures 3 and 4].

**Figure 5.**
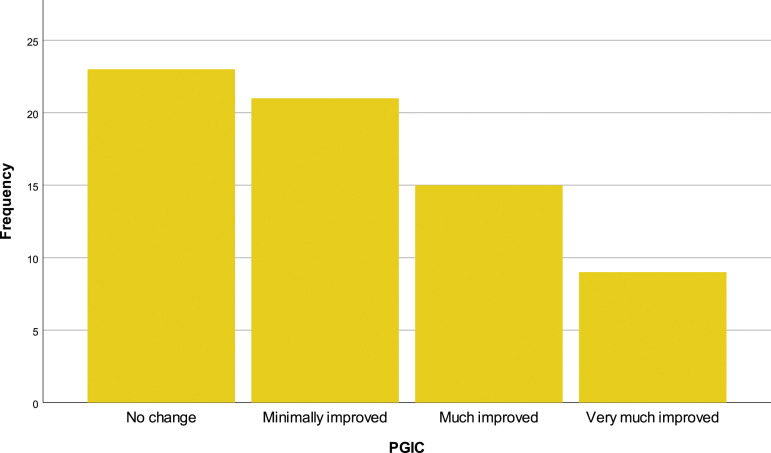
PGIC.

After the treatment, 33.8% (*N* = 23) of the patients did not feel improvements. In contrast, 30.9% (*N* = 21) felt minimally improved, 22.1 (*N* = 15) felt much improved and 13.2 (*N* = 9) felt very much improved [Figure 5]. 70.6% (*N* = 12) of patients with only one treatment reported no changes. All patients who underwent between 4 and 6 treatments claim improvement in pain. (Table 5) In post-surgical or traumatic pain and pain of unspecified aetiology, 67.4% (*N* = 31) and 76.9% (*N* = 10), respectively, report at least some improvement in pain. (Table 6)

**Table 5.** Relation between number of treatments and global perception of change.

PGIC	Number of treatments								Total
		1	2	3	4	5	6	7	
No change	*N* (%)	12 (70.6)	6 (27.3)	4 (30.8)	0 (0.0)	0 (0.0)	0 (0.0)	1 (50.0)	23 (33.8)
Minimally improved	*N* (%)	3 (17.6)	6 (27.3)	5 (38.5)	1 (25.0)	4 (66.7)	2 (50.0)	0 (0.0)	21 (30.9)
Much improved	*N* (%)	2 (11.8)	7 (31.8)	1 (7.7)	2 (50.0)	1 (16.1)	1 (25.0)	1 (50.0)	15 (22.1)
Very much improved	*N* (%)	0 (0.0)	3 (13.6)	3 (23.1)	1 (25.0)	1 (16.1)	1 (25.0)	0 (0.0)	9 (13.2)
Total		17	22	13	4	6	4	2	68

**Table 6.** Relation between aetiology and global perception of change.

PGIC	Aetiology							Total
		Amputation	DNP	Post-surgical or trauma	CINP	Post-herpetic	Other	
*No change*	*N* (%)	1 (100.0)	0 (0.0)	15 (32.6)	3 (75.0)	1 (33.3)	0 (0.0)	23 (33.8)
*Minimally improved*	*N* (%)	0 (0.0)	1 (100.0)	14 (30.4)	0 (0.0)	0 (0.0)	6 (46.2)	21 (30.9)
*Much improved*	*N* (%)	0 (0.0)	0 (0.0)	10 (21.7)	1 (25.0)	1 (33.3)	3 (23.1)	15 (22.1)
*Very much improved*	*N* (%)	0 (0.0)	0 (0.0)	7 (15.2)	0 (0.0)	1 (33.3)	1 (7.7)	9 (13.2)
*Total*		1	1	46	4	3	13	68

DNP: diabetic polyneuropathy, CINP: chemotherapy induced peripheral neuropathy.

**Figure 6.**
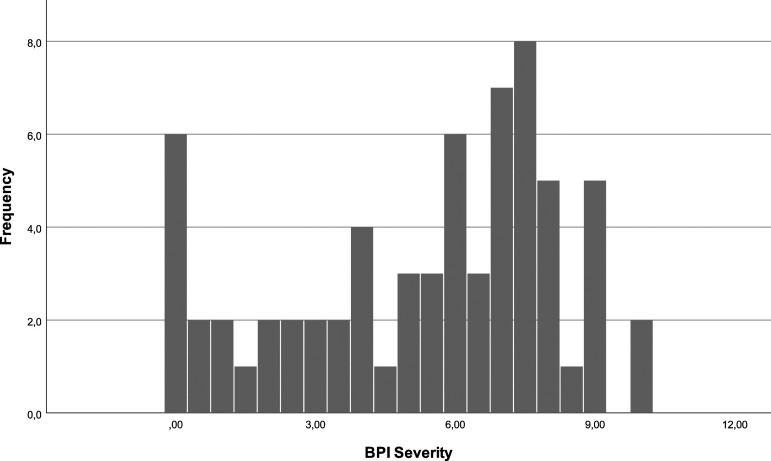
BPI severity.

**Figure 7.**
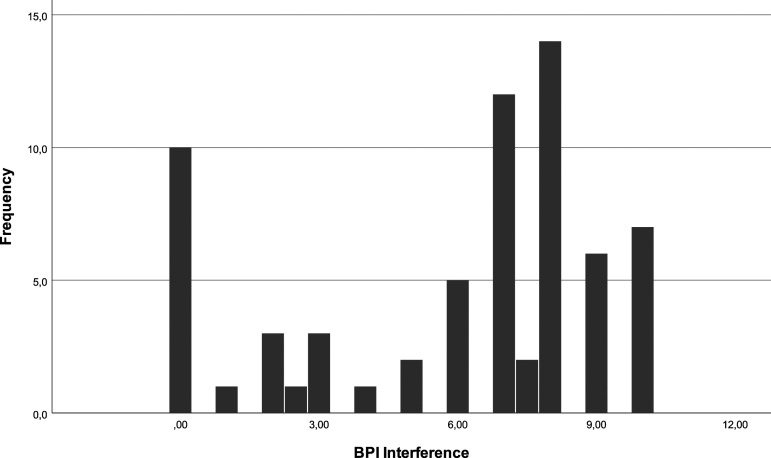
BPI interference.

In the week prior to the telephone survey, 9% (*N* = 6) of the patients did not experience pain. On a scale of 0 to 10, 25% of the patients affirm an intensity under 3, with 9% (*N* = 6) reporting no pain. 50% of patients report pain between 3 and 7.50, with a median of 6, and 25% over 7.50 [Figure 6]. Regarding pain interference the week before the survey, 14.9% (*N* = 10) affirm no interference. On a scale of 0 to 10, 25% report an interference under 3, 50% between 3 and 8 and 25% over 8 with a median of 7 [Figure 7].

**Figure 8.**
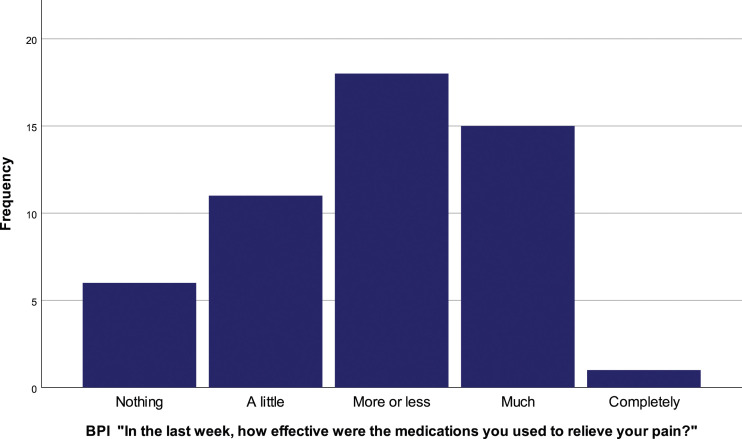
BPI “In the last week, how effective were the medications you used to relieve your pain?”

Only 2% (*N* = 1) of the patients affirm that current analgesics were completely effective in relieving pain the week before the study. 35.6% (*N* = 18) report more or less pain relief with medication, 29.4% (*N* = 15) report a lot of efficacy, 21.6% (*N* = 11) have little relief and 11.8% (*N* = 6) affirm no relief with medication [Figure 8].

**Figure 9.**
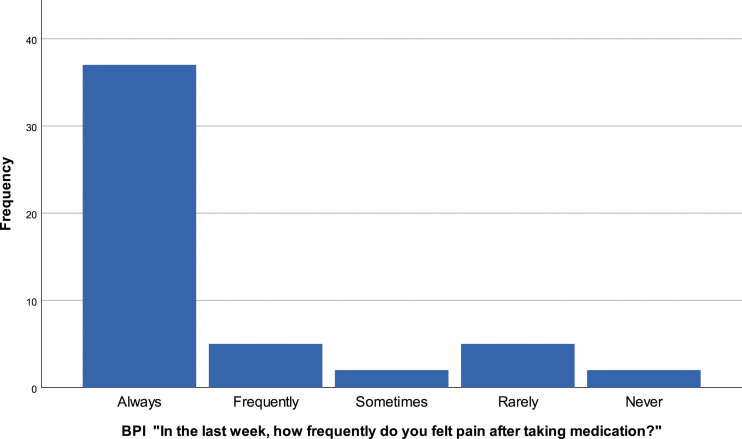
BPI “In the last week, how frequently do you felt pain after taking medication?”

The majority of patients 72.5% (*N* = 37) report always feeling pain, even with medication. 9.8% (*N* = 5) affirm that is very frequent to have pain after medication, 9.8% (*N* = 5) report that rarely have pain after taking analgesics, 3.9% (*N* = 2) say sometimes have pain after analgesics and only 3.9% (*N* = 2) never have pain after taking medication [Figure 9].

**Figure 10.**
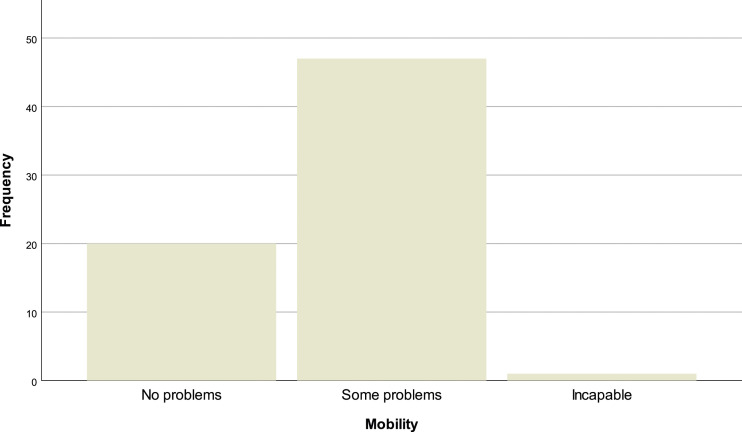
EQ-5D mobility.

**Figure 11.**
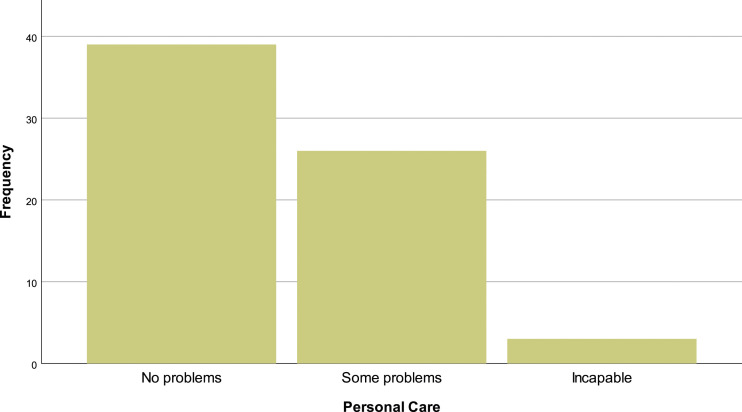
EQ-5D personal care.

**Figure 12.**
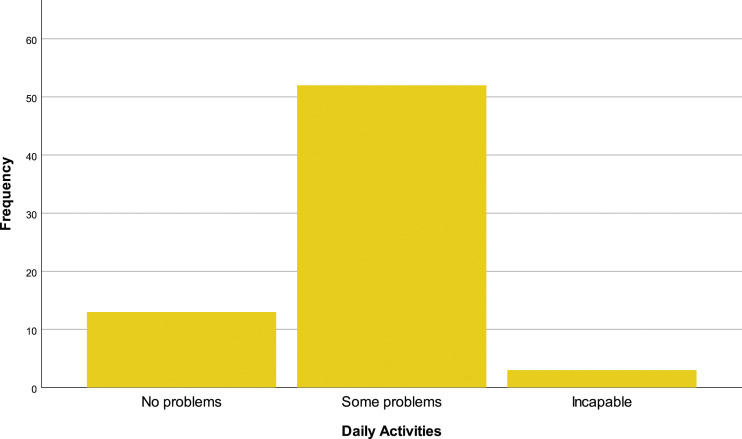
EQ-5D daily activities.

**Figure 13.**
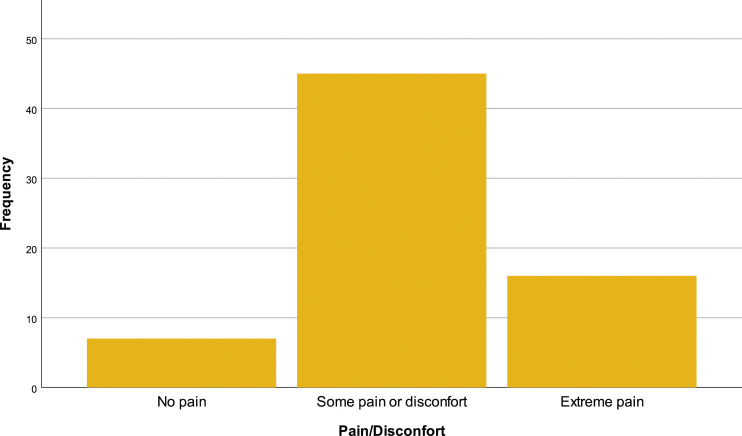
EQ-5D pain/disconfort.

**Figure 14.**
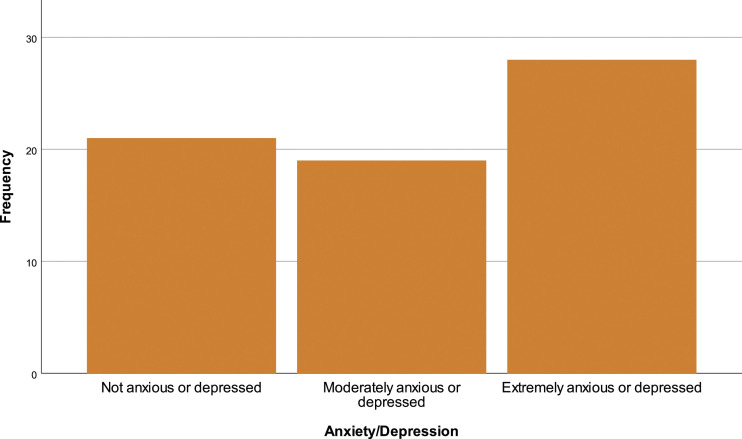
EQ-5D anxiety/depression.

In the EQ-5D-3L questionnaire, 69.1% (*N* = 47) of the patients reported having problems walking, 29.4% (*N* = 20) have no mobility problems and 1.5% (*N* = 1) are incapable of walking [Figure 10]. In personal hygiene, 57.4% (*N* = 39) affirm no limitations, 38.2% (*N* = 26) have limitations and 4.4% (*n* = 3) are incapable of doing their personal care by themselves [insert Figure 11]. Regarding daily activities, 76.9% (*N* = 32) reported some problems, 19.1% (*N* = 13) have no problems and 4.4% (*N* = 3) are incapable of doing their daily activities [Figure 12]. The majority, 66.2% (*N* = 45), affirm moderate pain or discomfort, 23.5% (*N* = 16) affirm extreme pain or discomfort and 10.3% (*N* = 7) report no pain or discomfort [Figure 13]. At last, 41.2% (*N* = 28) feel extremely depressed or anxious, 30.9% (*N* = 21) do not feel anxious or depressed and 27.9% (*N* =19) feel moderately anxious or depressed [Figure 14].

## Discussion

This study aims to evaluate the effectiveness of the topical application of capsaicin in peripheral neuropathic pain and its impact on patients’ quality of life. In this set of patients, a total of 66.2% reported pain improvement in the short term after treatment with capsaicin 8% patch. More specifically, 30.9% report a mild improvement, 22.1% to very much improved and 13.2% affirm great improvement. No patient reported a worsening in pain after the capsaicin 8% patch treatment. When discussing these results, it is important to have in mind that PGIC is a personal perception. The absence of an impression of change does not mean there were not any changes, for example in the area of pain instead of intensity.^29,31,37–39,42^ Moreover, some patients were submitted to treatment almost 10 years ago, meaning that is important to consider a possible memory bias.

Although we acknowledge that we present a small sample for some etiologies, it is important to note that 67.6% of the patients have post-surgery or trauma pain. Of this, 32.6% report no change in pain after treatment, but 67.4% have improvement in pain. Post-surgical pain is one of the most common causes of neuropathic pain.^43,47^ It is expected to continue to increase its frequency as the number of surgeries performed increases.^37^ However, the evidence for capsaicin treatment in chronic post-surgical neuropathic pain was scarce,^43^ until recently when various studies found a significant pain intensity reduction with capsaicin 8% patch treatment in this group of patients.^37,43,48–50^ Our study is in line with the previously published results.

As for the long-term response, in the week prior to the telephone survey, 6 patients reported no pain. However, the median of pain intensity was 6 and half of the patients referred to pain in an interval between 3 and 7.50. This means they maintain mild to moderate pain, although claiming improvement right after the treatment. This may suggest that the analgesic effect is stronger in the first weeks after treatment and therefore repeated treatments may be important to maintain the analgesic effect.^28,39^

The published information states that high doses of capsaicin, like the 8% patch, induce analgesia through the defunctionalization of nociceptive nerve fibres due to a localized structural ablation of TRPV1-expressing afferent terminals.^18,22–24,26,30^ However, this defunctionalization is reversible. The affected nerve fibres may regenerate, usually from 12(26) to 24(22, 25, 32) weeks after the treatment. This knowledge supports the hypothesis that repeated treatment over time is necessary for a long-term analgesic effect.^28,36^

Other than long-term effects, repeated applications may be necessary for better response. Multiple studies concluded that repeated applications led to higher response rates.^11,36,38,39,41^ Our results fall in line with this data given that no patient reported great improvement with only one application, but in the 49 patients that received 2 to 6 treatments, only 10 patients reported no changes in pain. Furthermore, repeated treatments can be essential to a specific group of patients denominated slower responders. In these patients, two or three treatments more than 8 weeks apart may be needed to induce the analgesic effect.^40^ Therefore, the absence of an initial response, as was the case with 12 of our patients, should not be a motive to not proceed with the treatment.^38,40^

When discussing repeated applications, it is important to consider tolerability. Although it was not evaluated in this study, during the telephonic survey, a few patients referred to local reactions like burning pain in the first hours to days after treatment. Among this set of patients, only one patient affirmed that would not want to repeat treatment due to intense burning pain.

Literature demonstrates that capsaicin 8% patch treatment is overall well tolerated given the fact that is absorbed in the skin with minimal systemic concentration minimizing systemic adverse effects.^11,29,36–39^ The most common side effects involve small transient elevation of blood pressure and local reactions like burning pain, pruritus and erythema at the application site.^18,22,23,51^ These reactions, especially the intense burning pain, are one of the barriers to the wider use of capsaicin treatment. There is no effective method to eliminate these side effects, but attenuation of pain is recommended.^18,22^ In most cases is used topical agents, paracetamol or nonsteroidal anti-inflammatory drugs to manage the pain.^12,22,29,37,51^ Another possibility is skin cooling. Placing ice pads on the area of the patch during treatment does not affect the capsaicin effect^18^ and has been found to attenuate burning pain, improve tolerance and diminish the need for rescue analgesia.^12,18,37,51^

Nevertheless, the low dose and transient side effects allow for treatment with capsaicin 8% patch to be well tolerated in repeated applications. Repeated treatment with capsaicin 8% patch for over 48(51) and 52(11, 36) weeks was well tolerated without increasing the frequency of adverse reactions and minimal risk of adverse effects on neurological function.^11,36,51,52^

Another crucial factor to consider besides tolerability is drug interactions. Our set of patients has an average age of 61.46 years old, meaning that the possibility of being poli-medicated or having liver or renal impairment should be taken into account. Moreover, chronic pain patients are frequently poli-medicated, no matter their age. As previously stated, topical capsaicin treatment has a low total systemic dose and it does not inhibit or induce cytochrome P450 enzymes, meaning it will not interfere with the metabolism of other drugs.^11,18,22,26,29,36,37,39^

Regarding the quality of life, 10 patients report no pain interference in quality of life. However, 50% of patients claim an interference between 3 and 8 on a scale from 1 to 10. The median in this variable was seven. It is important to note that the median value in BPI for interference is higher than the intensity. This shows that patients value the interference of pain in their quality of life over pain severity, proving that interference is an equally important variable as pain reduction when evaluating pain treatments.

The results in EQ-5D-3L show that the majority of patients have mobility limitations, problems in performing their daily activities and moderate pain or discomfort. Only in personal care do the majority of patients claim to not have limitations. 41.2% of patients report extreme anxiety or depression. Nevertheless, 30.9% report not having anxiety or depression associated with the pain. Studies using the EQ-5D-3L questionnaire affirm a meaningful improvement in quality of life when compared to pre-treatment values.^11,36,38,41,42^

The main limitation of this study is the fact that there were no baseline values of the applied questionnaires to establish a comparison. These values would be particularly relevant in the discussion of BPI and EQ-5D-3L results in order to evaluate the effect of capsaicin 8% patch treatment on pain intensity and quality of life. Another variable that could be taken into account is side effects prevalence to establish tolerability and patient willingness to repeat treatment. It would also be relevant to define the time between the last treatment and the telephonic survey, as several patients referred to improvement right after treatment but currently feel no effect at all of the analgesia provided by capsaicin. The study of this correlation would allow us to establish the period in which the treatments should be repeated to maintain the analgesic effect. In accordance with the available literature, repeated applications may be considered over 10–12 weeks.^28^ Lastly, the small number of patients included in this study can limit the application of these results to the general population.

## Conclusion

PNP is one of the most challenging diseases to treat. Several barriers arise in its therapeutic approach, from the poor effectiveness of drugs to their adverse effects that condition adherence to therapy. Furthermore, PNP has a significant negative impact on the patients’ quality of life, affecting their daily activities, relationships with others, sleep and mental health.

Capsaicin, in low doses, has been on the market for several decades as a compound in topical application. Recently, in the last 2 decades, the benefit of applying high doses of capsaicin in localized PNP has been explored.

Although this study has some limitations, it demonstrates that 66.2% of patients show at least a small notion of improvement with the application of the capsaicin 8% patch, proving that the 8% capsaicin patch is effective in PNP treatment. However, the analgesic effect seems to decrease with time and increase with the number of applications, establishing the need for repeated applications. The low systemic dose and few side effects mean that the treatment is generally well tolerated by patients. Due to the analgesic effect, capsaicin can provide an improvement in the quality of life of patients with PNP.
